# Supplementary material for: The genome sequence of the Garden Tiger, Arctia caja (Linnaeus, 1758) (Lepidoptera: Erebidae)
Source: Wellcome Open Res. 2025 Jul 30;10:385. [Version 1] doi: 10.12688/wellcomeopenres.24634.1 (PMC12552838; doi:10.12688/wellcomeopenres.24634.1)
Supplement: Peer review history

# Reviewer report: Reviewer response for version 1

**DOI:** 10.21956/wellcomeopenres.27148.r134206

**Published:** 2025-11-22

**Authors:** Sara Goodwin

**Affiliations:** 1 Cold Spring Harbor Laboratory, New York, USA

Review of “The genome sequence of the Garden Tiger, *Arctia caja* (Linnaeus, 1758) (Lepidoptera: Erebidae)

 ”

 The Darwin Tree of Life Project is an exciting genomic endeavor. This latest manuscript reads as a typical article that is part of this project and is concise.   This genome note reports a high-quality, chromosome-scale assembly for the Garden Tiger moth *Arctia caja*.   Both   PacBio HiFi Arima Hi-C were used to generate a phased assembly.  This assembly achied   99.0% complete genes for hap1  (BUSCO). Raw data and assemblies have been deposited at ENA under PRJEB78788

Are sufficient details of methods and materials provided to allow replication by others?

Yes

Is the rationale for creating the dataset(s) clearly described?

Yes

Are the datasets clearly presented in a useable and accessible format?

Yes

Are the protocols appropriate and is the work technically sound?

Yes

Reviewer Expertise:

Plant and animal genomics and sequencing technology

I confirm that I have read this submission and believe that I have an appropriate level of expertise to confirm that it is of an acceptable scientific standard.

# Reviewer report: Reviewer response for version 1

**DOI:** 10.21956/wellcomeopenres.27148.r134207

**Published:** 2025-10-24

**Authors:** Sivasankaran Kuppusamy

**Affiliations:** 1 Loyola College, Chennai, Tamil Nadu, India

The authors have performed a chromosome-level genome assembly of the Garden Tiger, *Arctia caja* (Linnaeus, 1758). They generated 160.49 Gb of genomic data using PacBio and Illumina sequencing platforms. The assembly resulted in two haplotypes with total lengths of 700.59 Mb (chromosome level) and 699.20 Mb, respectively. High-molecular-weight DNA was isolated using appropriate methods, and suitable software tools were employed for genome assembly and annotation.

**Comments on the manuscript**

 In the Background section, in the first sentence of the third line, the term “hind wings” appears as two separate words. It would be more appropriate to write it as “hindwings” (a single word) for consistency with standard entomological usage.

 The authors have produced 160.49 Gb of robust genome data. However, the numbers of protein-coding genes, non-coding genes, and gene transcripts have not been identified through genome annotation and are not listed in the table. Could the authors clarify the reason for this omission?

 The authors have used the full form of the genus name *Arctia* throughout the manuscript. The genus name should be written in full only at its first mention and may thereafter be abbreviated (e.g., *A. caja*).

 Above all, I confirm that the manuscript meets the necessary scientific standard and is suitable for indexing.

Are sufficient details of methods and materials provided to allow replication by others?

Yes

Is the rationale for creating the dataset(s) clearly described?

Yes

Are the datasets clearly presented in a useable and accessible format?

Yes

Are the protocols appropriate and is the work technically sound?

Yes

Reviewer Expertise:

Phylogenetic analysis of Noctuoidea moths using mitogenome analysis

I confirm that I have read this submission and believe that I have an appropriate level of expertise to confirm that it is of an acceptable scientific standard.

# Reviewer report: Reviewer response for version 1

**DOI:** 10.21956/wellcomeopenres.27148.r134205

**Published:** 2025-09-30

**Authors:** Will Nash

**Affiliations:** 1 The University of East Anglia, Norwich, Norwich, UK

A chromosome level assembly for the Lepidopteran *Artica caja* is presented. This assembly was presumably sequenced as part of Project Psyche? This is not explicitly stated in the Background and could be clarified?

 The assembly uses cutting edge methodologies. These are well documented and reproducible. PacBio sequencing is produced from the Revio platform, and this is scaffolded with Hi-C data. RNA-seq data is generated for annotation. Bioinformatic pipelines are well documented, and versions are provided.

 The assembly is 700.59 Mb over 108 scaffolds with an N50 of 24.69Mb. 99.63% of the assembly is assigned into 31 chromosome level scaffolds. The assembly is high quality: QV 66.6, 99.62 kmer completeness, and representing 99.0% of the lepidoptera_odb10 BUSCO set.

 Assemblies and raw data is available through ENA.

Are sufficient details of methods and materials provided to allow replication by others?

Yes

Is the rationale for creating the dataset(s) clearly described?

Yes

Are the datasets clearly presented in a useable and accessible format?

Yes

Are the protocols appropriate and is the work technically sound?

Yes

Reviewer Expertise:

Population Genomics, Genome Assembly

I confirm that I have read this submission and believe that I have an appropriate level of expertise to confirm that it is of an acceptable scientific standard.
